# 

**Published:** 1888-06-30

**Authors:** 


### CONTENTS.

**London Hospitals and "The Pay System"** - - 205

**Venus or Æsculapius?** - - - - - - 206

**The Doctor's Pulpit.**—A Question of Smoke - - - 207

**In and Out Among the Hospitals.**—King's College Hospital—City of London Hospital for Diseases of the Chest—The Suffolk General Hospital - - - - - 208

**The Hospitals Association** - - - - - 209

**An Old and New English Spa.**—Woodhall. By OUR SPECIAL COMMISSIONER - - - - - - 211

**Notes and News** - - - - - - - 212

**Everybody's Page** - - - - - - - 214

**Annotations.**—The Celt and the Teuton.—Certain Knaves and Certain Fools - - - - - - - 215

**Turning Over New Leaves.**—"The New Judgment of Paris."—"Posological and Therapeutic Tables."—Magazines of the Month - - - - - - - 216

**An Ishmaelite.** By LESLIE KEITH, Author of "The Chilcotes," "East and West," etc. Chap XII.—Effects of a Library - 217

**General Practitioners and Paying Patients.** By DAVID WALSH, L.R.C.P. and S., Edin. - - - - 219

**Amusements with Profit for all Ages.**—For Men and Women.—Children's Corner.—Puzzles, etc. - - - 220

**EXTRA SUPPLEMENT.—THE NURSING MIRROR.**

En Passant.—Women as Apothecaries.—The Women's League.—Nurses in Many Lands.—Through Training.—One of the Olden Time.—The Sheffield Strike.—The Art of Resting - xlix
Original Articles.—Nursing in New York - - - - - l
Everybody's Opinion.—Nurses and Patients: A Retort from Manchester.—An Answer from Devon.—The Nurse Speaks.—A Probationer's View.—Presence of Mind *v.* Panic—A Reply - li
Appointments.—Honours and Presentations.—The Nurses' Bookshelf: "Hints for Matrons and Pupils in Cottage Hospitals."—Vacancies.—Notes and Queries - - - lii

